# Retention of an Endosymbiont for the Production of a Single Molecule

**DOI:** 10.1093/gbe/evae075

**Published:** 2024-04-05

**Authors:** Arkadiy I Garber, Andrés Garcia de la Filia Molina, Isabelle M Vea, Andrew J Mongue, Laura Ross, John P McCutcheon

**Affiliations:** Biodesign Center for Mechanisms of Evolution and School of Life Sciences, Arizona State University, Tempe, AZ, USA; Institute of Ecology and Evolution, University of Edinburgh, Edinburgh, UK; Institute of Ecology and Evolution, University of Edinburgh, Edinburgh, UK; Institute of Ecology and Evolution, University of Edinburgh, Edinburgh, UK; Department of Entomology and Nematology, University of Florida, Gainesville, FL, USA; Institute of Ecology and Evolution, University of Edinburgh, Edinburgh, UK; Biodesign Center for Mechanisms of Evolution and School of Life Sciences, Arizona State University, Tempe, AZ, USA; Howard Hughes Medical Institute, Chevy Chase, MD, USA

**Keywords:** nutritional symbiosis, endosymbionts, mealybugs, genome reduction, evolutionary entrenchment

## Abstract

Sap-feeding insects often maintain two or more nutritional endosymbionts that act in concert to produce compounds essential for insect survival. Many mealybugs have endosymbionts in a nested configuration: one or two bacterial species reside within the cytoplasm of another bacterium, and together, these bacteria have genomes that encode interdependent sets of genes needed to produce key nutritional molecules. Here, we show that the mealybug *Pseudococcus viburni* has three endosymbionts, one of which contributes only two unique genes that produce the host nutrition-related molecule chorismate. All three bacterial endosymbionts have tiny genomes, suggesting that they have been coevolving inside their insect host for millions of years.

**Significance**

Nutritional endosymbionts synthesize—or contribute to the synthesis of—key metabolites such as essential amino acids and vitamins for their host organism. These nutrients are required by hosts because of their limited diets, which in the case of mealybugs are limited strictly to plant phloem sap. Genome sequencing of insect endosymbionts has shown that their genomes can be very small, encoding a few genes outside of core bacterial processes and nutrient provisioning. Here, we highlight an example that has taken this reductive process to the extreme: a mealybug endosymbiont contributes only a single unique essential compound to the symbiosis.

## Introduction

Sap-feeding insects form long-term endosymbioses with bacteria or fungi to supplement their diets with essential amino acids and vitamins (Baumann 2005). Bacteria that form endosymbioses undergo stereotyped and sometimes extreme genome reduction during coevolution with their insect hosts (McCutcheon and Moran 2011). Endosymbionts are sometimes supplemented or replaced by new bacterial or fungal symbionts (Koga and Moran 2014; Husnik and McCutcheon 2016; Matsuura et al. 2018; Dial et al. 2021). In mealybugs (Hemiptera: Pseudococcidae), as in other related insects (Bennett and Moran 2013; Oakeson et al. 2014; Mao and Bennett 2020), symbiont replacement and supplementation have occurred multiple times, resulting in a diversity of symbiont types and ages across species (Husnik and McCutcheon 2016).

For example, in the handful of mealybug species with available genomic data, numerous bacterial symbionts in the *Sodalis* genus have been found whose genomes range in size over an order of magnitude, from 3.7 Mb (Garber et al. 2021) to 0.35 Mb (Husnik and McCutcheon 2016). It is thought that this variation in genome size reflects variation in endosymbiont age: newly established endosymbionts tend to have larger genomes, and endosymbionts that have had long associations tend to have smaller genomes (Moran 1996; Andersson and Kurland 1998; Andersson and Andersson 1999; Wernegreen 2002; Moran et al. 2009; Wolf and Koonin 2013; Oakeson et al. 2014).

In most sequenced mealybugs, a single *Sodalis* endosymbiont resides within the cytoplasm of another bacterial endosymbiont, *Tremblaya princeps* (von Dohlen et al. 2001). There has been one report of a mealybug with two intra-*Tremblaya* endosymbionts, both with large genomes and likely recently acquired (Garber et al. 2021). Here, we report a similar three-way endosymbiosis, but where all symbionts have highly reduced genomes and so we infer that they have been coevolving with their host insect for millions of years. Remarkably, one endosymbiont provides only one unique nutrition-related molecule to the symbiosis.

## Results and Discussion

### Endosymbiont Genome Assembly and Binning

Hybrid assembly of endosymbiont contigs using PacBio and Illumina reads resulted in four circular-mapping contigs, two of which (754,563 and 281,389 bp) are affiliated with the *Sodalis* group within Gammaproteobacteria. The other two circular contigs (123,124 and 20,943 bp) belong to *T. princeps*. Combined, the two *Tremblaya* contigs add up to the typical size of *Tremblaya's* genome (144 kb) from other mealybug species (Husnik and McCutcheon 2016). It is unclear how *Tremblaya's* genome has fragmented into two circles, but genome instability is not uncommon in endosymbionts (Van Leuven et al. 2014; Campbell et al. 2015; Campbell et al. 2017) and mitochondria (Palmer and Shields 1984; Vlcek et al. 2011; Shao et al. 2012; Wu et al. 2015; Shao et al. 2017). Read mapping revealed that both gammaproteobacterial contigs have similar but distinct read coverages (81× and 104×). *Tremblaya* has a much higher read coverage (1798×) and likely maintains many copies of its genome, as reported in the *Tremblaya* symbiont of the long-tailed mealybug, *Pseudococcus longispinus* (Garber et al. 2021) and in the obligate intracellular symbionts of other insects (Komaki and Ishikawa 1999, Woyke et al. 2010, Van Leuven et al. 2014).

### *Pseudococcus viburni* Harbors Two Ancient *Sodalis*-Related Endosymbionts

**Fig. 1.**
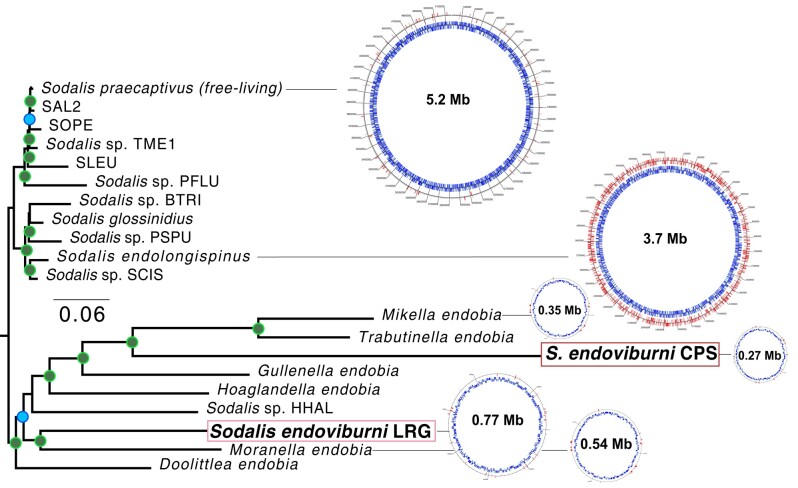
Phylogenomic tree showing the relationship of the two *P. viburni* gammaproteobacterial endosymbionts (highlighted within dark and light red boxes) with other members from the *Sodalis* clade. Genome maps from select *Sodalis*-related endosymbionts, as well as the free-living *Sodalis praecaptivus*, are shown. Numbers inside each genome map show the size of the genome in megabases (million bases); genome maps are divided into two tracks, with the blue inner track showing the locations of protein-coding genes, and the other red track shows the locations of predicted pseudogenes. Nodes with 99% or more support are designated with filled green circles. Nodes with support values between 80% and 98% are colored blue. Nodes with less than 80% support are unlabeled.

Each *Sodalis*-related contig encodes its own complete set of ribosomal proteins, tRNA genes, and rRNA genes (supplementary fig. S1, Supplementary Material online). The larger 755-kb contig encodes two copies of the rRNA operon (supplementary fig. S2, Supplementary Material online). A phylogenomic tree (Fig. 1) supports the presence of two species of *Sodalis* symbionts, with one endosymbiont (755 kb) clustering with *Moranella endobia* (hereafter, *Moranella*) (McCutcheon and von Dohlen 2011) and the other (281 kb) branching off from the phylogenetic cluster that encompasses *Mikella endobia* (Husnik and McCutcheon 2016) and *Trabutinella endobia* (Szabó et al. 2017). The similar read coverage depth of each *Sodalis*-related endosymbiont suggests that cells from both symbiont species are present at similar abundances.

The two *Sodalis* endosymbionts have highly reduced and gene-dense genomes, with relatively few pseudogenes (<10%). These features, along with long-branch lengths in the phylogenomic tree, suggest that both *Sodalis*-related endosymbionts are ancient (Moran 1996; Wernegreen 2002, McCutcheon and Moran 2011).

### Naming of the Novel *Sodalis*-Related Symbionts

We propose the name *Candidatus Sodalis endoviburni* LRG (hereafter, *S. endoviburni* LRG) for the *Sodalis*-allied organism with the larger genome (LRG meaning large) and *Candidatus S. endoviburni* CPS (hereafter, *S. endoviburni* CPS) for the *Sodalis*-allied organism with the smaller genome (CPS reflecting that all this organism seems to contribute to the symbiosis is carbamoyl phosphate synthesis; see the next section for a description of this genome).

### Carbamoyl Phosphate Synthase: *S. endoviburni* CPS's Only Contribution to the Symbiosis

**Fig. 2.**
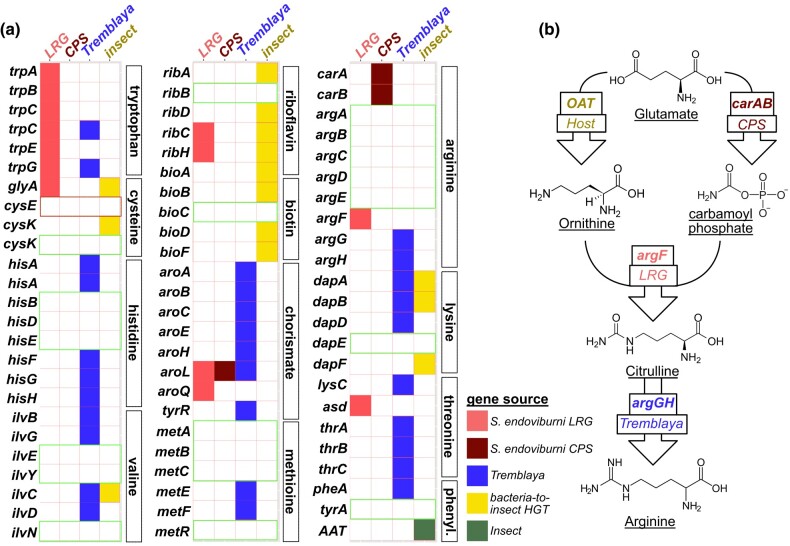
a) The presence/absence matrix showing the partitioning of biosynthetic pathway components across the *P. viburni* symbionts and host. The two *Sodalis*-related endosymbionts are denoted simply with LRG and CPS. b) Diagram of arginine biosynthesis is *P. viburni*, showing the sole role of *S. endoviburni* CPS in generating the carbamoyl phosphate that is essential of the synthesis of citrulline, a precursor of arginine. Green boxes represent pathway components that are missing in both *P. viburni* and *P. citri* mealybugs; red boxes represent pathway components that are missing only in *P. viburni*.

To examine nutritional contributions and metabolic complementarity between the two *Sodalis* endosymbionts of *P. viburni*, we screened both genomes, along with *Tremblaya* and the host's genome, for pathways relevant to amino acid and vitamin biosynthesis (Baumann 2005; Douglas 2006). We found that genes for these pathways are mostly retained on the genomes of *S. endoviburni* LRG, *Tremblaya*, and the host (Fig. 2a). The nuclear genome of *P. viburni*, like the closely related mealybugs *P. longispinus* and *Planococcus citri*, encodes numerous bacterial genes (acquired via horizontal gene transfer) that seem to complement genes missing from the bacterial symbiont genomes (Husnik and McCutcheon 2016; Bublitz 2019). Our screen identified the same horizontal gene transfers (HGTs) in *P. viburni* that were previously reported in the citrus mealybug *P. citri* (Husnik et al. 2013), suggesting these HGT events occurred prior to the split between *Pseudococcus* and *Planococcus*. Surprisingly, *S. endoviburni* CPS seems to only contribute three genes related to the biosynthetic pathways for essential amino acids: the small subunit of carbamoyl phosphate synthase (*carA*), the large subunit of carbamoyl phosphate synthase (*carB*), and shikimate kinase II (*aroL*). While *aroL* is essential for the synthesis of chorismate and subsequently a number of aromatic amino acids, it is also present in the genomes of *S. endoviburni* LRG and *Tremblaya*. It thus appears that the only unique nutritional contribution from *S. endoviburni* CPS is carbamoyl phosphate (from *carAB*), used in the production of the essential amino acid arginine (Fig. 2b).

While *S. endoviburni* CPS represents the smallest genome within the *Sodalis* clade of symbionts, it is not the smallest symbiont genome sequenced so far. Smaller still are the symbionts of some sap-feeding leafhoppers, which have bacterial endosymbionts with genomes as small as about 100 kb, encoding more than three genes to enable to biosynthesis of essential metabolites from the insects’ sugar-based diet (Bennett and Moran 2013). Two other examples are similar to the level of specialization we report here for *S. endoviburni* CP. The first is the ancient symbiont *Stammera* of the plant-feeding leaf beetle, which only encodes a few genes required for the breakdown of pectin (Salem et al. 2017). The second is a case in which an endosymbiont genome appears to retain no symbiotic genes at all but rather seems to have eroded to the point of being nutritionally useless and likely destined for replacement (Manzano-Marín et al. 2018). Because the genes for the key nutritional molecule carbamoyl phosphate only exist on *S. endoviburni* CP, we expect that this endosymbiont is currently safe from extinction.

## Methods

### Insect Rearing

We used mealybugs from a colony reared to study the transmission of a selfish B chromosome (Vea et al. 2021). In brief, we initially obtained mealybugs from a glass house in the Royal Botanic Gardens of Edinburgh in Scotland; from these insects, we established a laboratory colony fed on sprouting potatoes at 25 °C on a 16-h light/8-h dark cycle.

### Sequencing and Assembly

Illumina and PacBio sequence reads were obtained from and processed as described in Vea et al. (2021). Illumina reads were quality trimmed using Trimmomatic v0.36 (minimum length = 36 bp, sliding window = 4 bp, and minimum quality score = 15 [ILLUMINACLIP:TruSeq3-PE:2:30:10 LEADING:3 TRAILING:3 SLIDINGWINDOW:4:15 MINLEN:36]) (Bolger et al. 2014). PacBio and Illumina reads were then assembled using Canu v1.6 (default parameters; Koren et al. 2017), resulting in 2,787 contigs and 440,161,839 bases. Contigs that appeared to be bacterial were extracted from the assembly using the SprayNPray software (Garber et al. 2022), and these putative endosymbiont contigs were then used to recruit Illumina and PacBio reads. Mapping of Illumina reads was carried out using Bowtie2 v2.3.4.1 (Langmead and Salzberg 2012). Mapping of PacBio reads was done with BLASR v5.1 (Chaisson and Tesler 2012). Using Unicycler v0.4.8 (default parameters; Wick et al. 2017), we then performed a hybrid assembly of the putative endosymbiont-affiliated PacBio and Illumina reads.

### Phylogenomic Analysis

Phylogenomic analyses were carried out using GToTree v1.5.38 (Lee 2019). Phylogenomic tree construction was carried out in RaxML, with 100 bootstraps (-N 100), the PROTCAT model for amino acid substitution, and the BLOSUM 62 amino acid matrix (-m PROTCATBLOSUM62) (Stamatakis 2014).

### Functional Annotation and Pseudogene Identification

We annotated each endosymbiont genome using Prokka (Seemann 2014), which also predicted genes and open reading frames (ORFs) using a variety of software, including Prodigal (Hyatt et al. 2010) and RNAmmer (Lagesen et al. 2007). Protein-coding genes were also annotated using the GhostKOALA annotation server (Kanehisa et al. 2016). Pseudogenes were identified using the software Pseudofinder (Syberg-Olsen et al. 2022). Annotation data were consolidated with the pseudogene predictions and organized in biosynthetic pathways using a semiautomated approach, which included custom Python scripts and visual inspection.

We identified putative bacteria-to-insect HGTs using the SprayNPray software (Garber et al. 2022) combined with previously published genomes (Husnik and McCutcheon 2016). Briefly, SprayNPray identified eukaryotic contigs using a combination of metrics, including contig length, coding density, and GC content. ORFs from eukaryotic contigs were then compared against NBIC's nonredundant database of proteins using DIAMOND (Buchfink et al. 2021), and the top 100 matches were evaluated. ORFs that recruited mostly (>50%) bacterial homologs were flagged as potential HGTs.

## Supplementary Material

evae075_Supplementary_Data

## Data Availability

Genomic data from the *Pseudococcus viburni* mealybugs was obtained from the following BioProject number: PRJEB47083, which was initially made available by Vea et al. (2021). Genome sequences and annotation data corresponding to *P. viburni* endosymbionts are available via FigShare: https://doi.org/10.6084/m9.figshare.24945384.v1.
