# Supplementary material for: Retention of an Endosymbiont for the Production of a Single Molecule
Source: Genome Biol Evol. 2024 Apr 5;16(4):evae075. doi: 10.1093/gbe/evae075 (PMC11032189; doi:10.1093/gbe/evae075)
Supplement: evae075_Supplementary_Data [file evae075_supplementary_data.pdf]

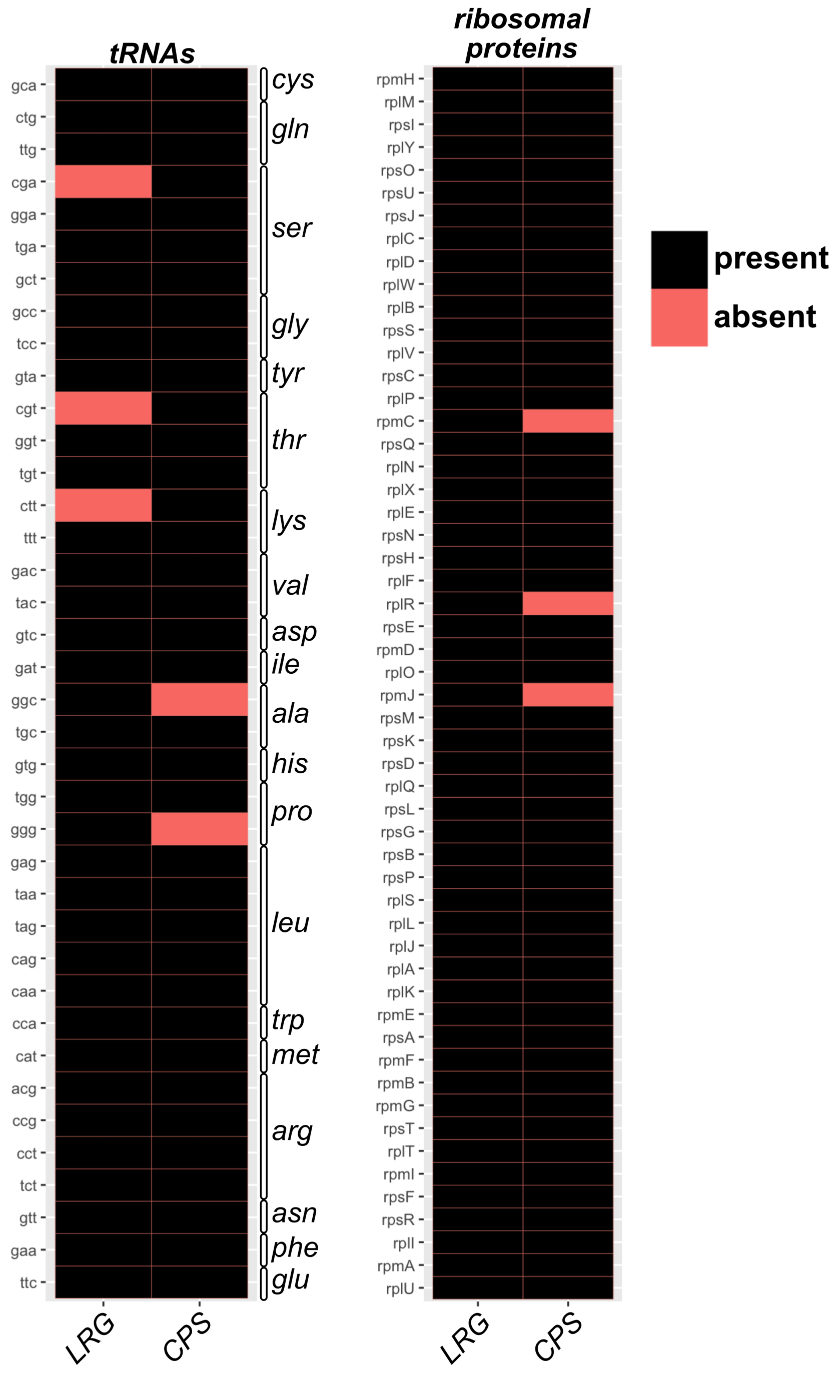

Supplemental Figure 1: Presence/absence matrix showing the distribution of tRNAs and ribosomal proteins in the two Sodalis-related endosymbionts of P. viburni.

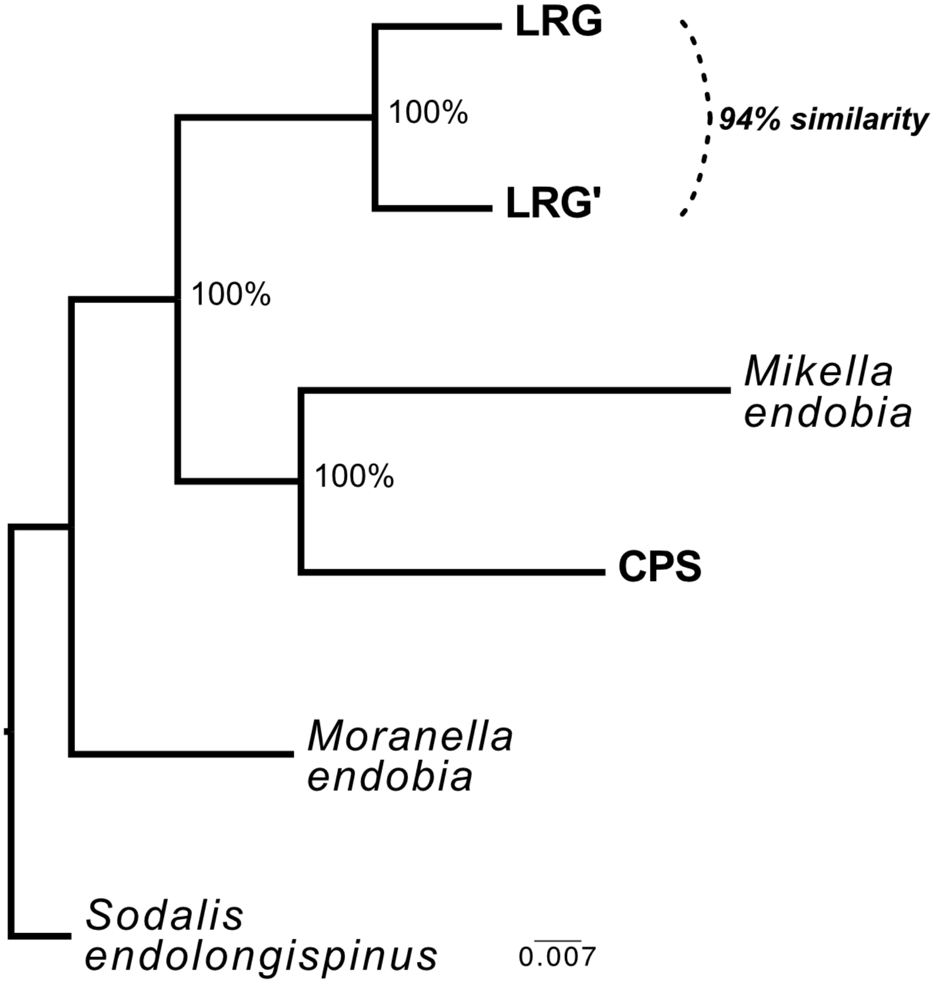

Supplemental Figure 2: A phylogenetic tree based on a concatenated alignment of the 5S, 23S, and 16S rRNA genes. The two copies of the 16S gene encoded by S. endoviburni LRG are denoted by LRG and LRG'
